# 

**Published:** 1891-02-14

**Authors:** 


[illegible] would have to be preferred to the Extract[illegible] for it would contain all the nutritive constituents [illegible]

before stated that in pre- [illegible]paring the Extract of Meat, the Albuminous principles remain in the residue, they are [illegible]ost to nutrition, and this is certainly a great disadvantage."

"BOVRIL" contains [illegible]the albumen and fibrine in the most perfect possible form, and to

[illegible] know the requirements of the human system and the constituents of food, it will be apparent that this albumen and

